# HOW CLINICAL SETTING AND EXPERIENCES INFLUENCE NURSING STUDENTS’ ATTITUDES TOWARD OLDER ADULTS

**DOI:** 10.1093/geroni/igz038.2783

**Published:** 2019-11-08

**Authors:** Susan Hovey

**Affiliations:** Illinois State University, Normal, Illinois, United States

## Abstract

This study is significant because older healthcare consumers continue to rise with estimations that nearly 72.1 million persons in the United States will be over the age of 65 by 2030. A fundamental question remains, will the future nursing workforce possess the attitude and knowledge to competently provide age-friendly care to older adults. The aim of this study explores how clinical setting, previous experiences with older adults, and previous work experiences in long-term care settings influence the attitudes of first year prelicensure nursing students toward this population. Six baccalaureate nursing schools from a Midwest state in the United States participated in this descriptive, cross-sectional, correlational study. One hundred and nine participants who completed their first clinical experience participated in the study. An understanding of this experience may provide nurse educators with insight into how to design clinical learning activities so nursing students’ acquire interest in care of older adults.

